# Supplementary material for: MetaRibo-Seq measures translation in microbiomes
Source: Nat Commun. 2020 Jun 29;11:3268. doi: 10.1038/s41467-020-17081-z (PMC7324362; doi:10.1038/s41467-020-17081-z)
Supplement: Supplementary file 10 — Supplementary Data 7 [file 41467_2020_17081_MOESM10_ESM.zip › File2/Confidence_High_Taxonomy/351033_out.krona.html]

Javascript must be enabled to view this page.

members
magnitude
magnitudeUnassigned
count
unassigned
taxon
rank

351033\_out

5

5
superkingdom
2

5
phylum
976

5
class
117743

5
200644
order

5
species
2021391

SRS043768\_contig\_number\_contig-100\_1334.193467SRS050752\_contig\_number\_contig-100\_1552.187228SRS1041039\_contig\_number\_contig-100\_904.162070SRS1041090\_contig\_number\_contig-100\_1607.55450SRS1041137\_contig\_number\_contig-100\_1840.171535
